# LESS hysterectomy through a bluntly created 11 mm incision

**DOI:** 10.4274/jtgga.galenos.2020.2020.0028

**Published:** 2021-02-24

**Authors:** Greg Marchand, Ali Azadi, Sienna Anderson, Stacy Ruther, Sophia Hopewell, Giovanna Brazil, Katelyn Sainz, Hannah Wolf, Alexa King, Jannelle Vallejo, Kelly Ware, Kaitlynne Cieminski, Anthony Galitsky

**Affiliations:** 1 Department of Minimally Invasive Surgery, The Marchand Institute for Minimally Invasive Surgery, Mesa, United States of America; 2 Department of Urogynecology, Star Urogynecology Advanced Pelvic Health Institute for Women, Arizona, United States of America; 3 Washington University of Health and Science School of Medicine, San Pedro, Belize; 4 International University of Health Sciences School of Medicine, Basseterre, Saint Kitts and Nevis

**Keywords:** Hysterectomy, single port, LESS, laparoendoscopic single site surgery, robotic hysterectomy, laparoscopic hysterectomy, laparoscopy

## Abstract

In the field of minimally invasive surgery, there is a constant drive to devise and execute the most minimally invasive surgeries possible. By the very nature of laparoscopy and robotic surgery, what one can accomplish with several ports of a given size will invariably be studied and attempted with fewer ports and with ports of smaller sizes. After researching the literature, we were not able to find any single port hysterectomies performed through a port size of smaller than 15 mm. We were able to perform, described here, a technique for performing laparoscopic hysterectomy through a single port of only 11 mm in diameter. We illustrate the technique in the accompanying video and believe the technique to be safe and reproducible.

## Introduction

Unlike other specialties which are defined by the general field of medicine they pertain to, “minimally invasive surgery” itself can be understood as a challenge to its practitioners, its very name encouraging them to pursue a more minimally invasive approach. The specific issue we sought to address here was attempting the most minimally invasive, single-port hysterectomy ever performed, while still performing meaningful laparoscopic visualization of the abdomen and with the expectation to be able to realistically operate in the abdomen from a laparoscopic approach. This meant that we specifically did not wish to perform a procedure that one could consider to be a laparoscopy then followed by vaginal hysterectomy, and desired meaningful laparoscopic access to deal with issues such as adhesions, mobilization of the bladder flap, or performing a bilateral salpingo-oophorectomy without significant vaginal assistance. After researching the literature, we were not able to find any single port hysterectomies performed through a port size of smaller than 15 mm (1). The authors also wished to exclude cases with no significant pathology or adhesive disease, as the purpose of describing the technique is to show that the technique can be used in many challenging situation, not to demonstrate the technique can be successful on the easiest of hysterectomies. All authors strongly contend that hysterectomies that can be performed through a completely vaginal technique should be, and that a vaginal hysterectomy, or zero port hysterectomy, is superior to laparoscopic hysterectomy, if it can be accomplished (2).

Multiple authors have documented the feasibility of single incision laparoscopic hysterectomy (3).  Many authors have commented that the idea, although novel, does not significantly improve intra-operative pain, recovery or surgical cosmesis (4). The most commonly used system is a robotic assisted single port system. All systems, to the knowledge of the authors, require incisions greater than 15 mm in the umbilicus (5,6). We examined different single port systems and combined available instrumentation to create a feasible, repeatable technique for performing a laparoscopic single site hysterectomy using only an 11 mm umbilical incision that is created with a blunt laparoscopic trochar. We have explained the technique in a video for reproducibility.

## Objective

**Figure 1:**
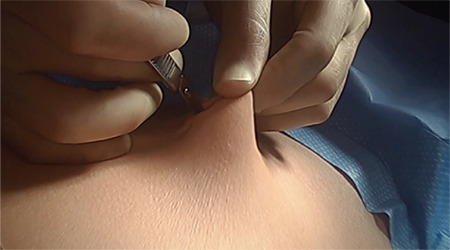
An 11 mm incision is made at the bottom of the patient’s umbilicus with an 11-blade scalpel

**Figure 2:**
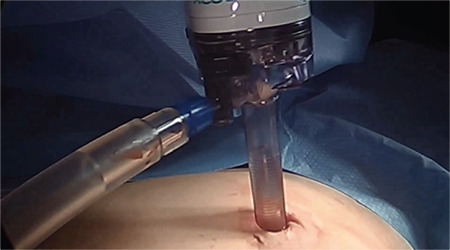
A blunt 11 mm laparoscopic trochar is utilized to make the entry into the abdominal cavity, in order to avoid a sharp dissection into the abdomen which would result in a larger fascial footprint

**Figure 3:**
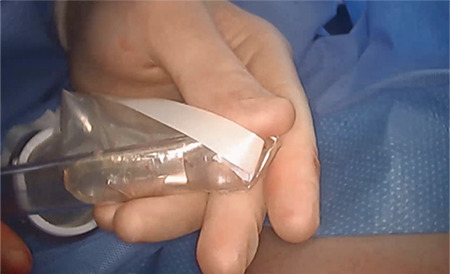
A multiport device is then loaded into the introducer, for insertion into the abdomen

**Figure 4:**
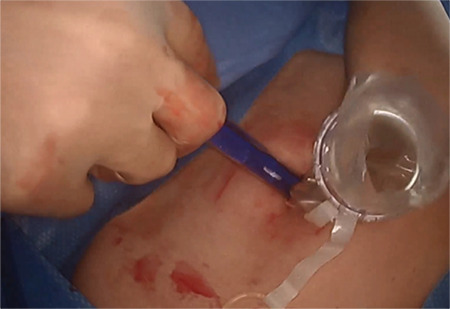
The multiport device is inserted through the abdominal incision after withdrawing the 11 mm blunt trochar. This ensures the incision width will not exceed 11 mm and has been created by blunt entry, which further decreases the chance of postoperative hernia

**Figure 5:**
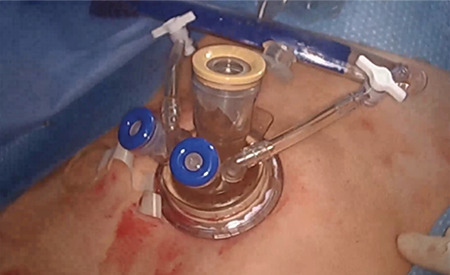
The multipart device is in place and the laparoscopic hysterectomy can proceed with one or two instruments in addition to the 5 mm laparoscope

**Figure 6:**
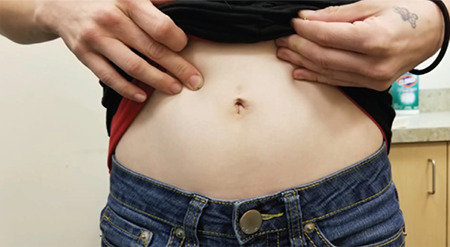
The patient’s abdomen at a visit four weeks after surgery. No scars are visible

**Figure 7:**
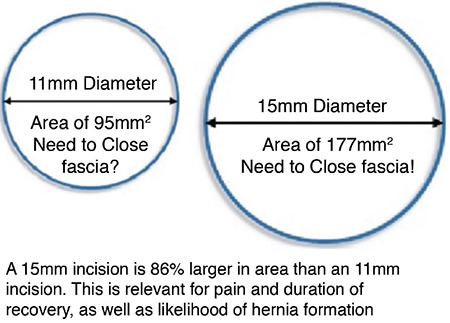
Secondary to the fact that laparoscopic incisions are stretched into a circular shape by the penetrating instrumentation, even a small decrease in the size of a fascial incision will greatly decrease the area of the opening that can pass through that incision. This figure compares the large jump from an area of 95 mm^2^ to 177 mm^2^ when increasing the umbilical incision by only 3 mm

We devised a technique for laparoscopic single port hysterectomy based on the concept that a bluntly created incision would be less likely to herniate than a sharply created incision. Therefore, after creating the initial skin incision with an 11-blade scalpel, (Figure 1) rather than perform an open dissection that would result in a large incision and a much larger fascial footprint, we then place an 1 mm blunt laparoscopic trochar (Figure 2) into the incision after insufflating with a veress needle. The multi-port device is then loaded into its introducer, (Figure 3) and is inserted into the abdominal cavity after removing the 11 mm port from the umbilical incision (Figure 4). The multi-port device can then be installed and actively utilized to perform the hysterectomy through only an 11 mm incision (Figure 5). Following this, the uterine pedicles are divided with a bipolar power coagulation and division device, and the circumferential colpotomy is made with a monopolar cautery set to 30 watts of coagulating current with a laparoscopic hook extender. The vaginal cuff is sewn from the vaginal approach and ovaries and tubes are removed after removal of the uterus. The patient returned foru weeks post-operatively and no scars were visible (Figure 6). We believe this technique to be significantly different from any previously described techniques because of the usage of an 11 mm blunt trochar to create the umbilical incision. This creates a reproducible footprint in the fascia that should be identical and reproducible, regardless of circumstances. By keeping the incision small and created bluntly we believe the risk of postoperative herniation has been minimized (Figure 7).

## Design

A narrated video demonstration of the surgical procedure (Canadian Task Force Classification III). We developed a novel method for performing laparoscopic hysterectomy through a single 11 mm incision that was created with a blunt trochar. The most novel aspects of our procedure involve the placement of a multiport manipulator device through a small, 11 mm incision created by an 11 mm blunt trochar. It is our belief that the small size of this blunt trochar likely makes fascial closure unnecessary, although it is still recommended by the authors.

## Interventions

A 32-year-old woman with endometriosis, adenomyosis and chronic pelvic pain with recurrent ovarian cysts presented for laparoscopic hysterectomy with bilateral salpingo-oophorectomy. The patient had previously tried more conservative surgeries and medical treatments, including a six-month course of luprolide acetate and multiple surgeries for fulgaration of endometriosis. The patient completed her desired childbearing and requested definite treatment. The patient had a history of prior bilateral salpingectomy and one prior cesarean section. The patient had confirmed endometriosis at previous laparoscopic exploration, and was suspected to suffer from adenomyosis, based on cyclic pain and pain that seemed to originate from the uterus with gentle palpation with the vaginal ultrasound probe. Patient was extensively counseled to the risks of bilateral salpingo-oophorectomy and offered more conservative surgical options including hysterectomy without bilateral salpingo-oophorectomy. The patient refused more conservative treatments, citing her fear of the necessity of future surgeries for endometriosis or ovarian cysts, the desire for definitive treatment of endometriosis, as well her fear of ovarian cancer in the future, despite there being no family history. Patient politely refused BRCA testing, citing that it would not influence her decision for bilateral salpingo-oophorectomy. The total operative time was 38 minutes, and the estimated blood loss was 100 cc. The patient was discharged 18 hours after surgery and the recovery was uneventful. The final pathology report showed endometriosis and adenomyosis.

## Conclusion

Our described technique is a feasible, reproducible procedure for hysterectomy and may improve cosmesis and postoperative pain over traditional laparoscopic and single port techniques.

**https://www.doi.org/10.4274/jtgga.galenos.2020.2020.0028.video1**
